# Effects of inorganic and organic selenium sources on the growth performance of broilers in China: A meta-analysis

**DOI:** 10.1515/biol-2021-0007

**Published:** 2021-01-20

**Authors:** Chunbo Wei, Xiuwei Lin, Ying Zhang, Xuanchen Wan, Haotong Wu, Tao He, Kai Bi, Changping Wang

**Affiliations:** Heilongjiang Provincial Key Laboratory of Efficient Utilization of Feed Resources and Nutrition Manipulation in Cold Region, Department of Animal Science, College of Animal Science and Veterinary Medicine, Heilongjiang Bayi Agricultural University, Daqing, 163319, China; Department of Plant Quarantine, College of Life Science, Jiamusi University, Jiamusi, China

**Keywords:** broiler, growth performance, inorganic selenium, organic selenium, meta-analysis

## Abstract

This study aims to investigate the effects of different selenium (Se) sources on the growth performance of Chinese broilers and provide a scientific rationale for adding Se additives to broiler feed. Relevant studies that meet standard inclusion criteria were identified and extracted from China National Knowledge Infrastructure and Wanfang and Chinese Scientific Journal (VIP) databases. A total of 9 studies with 539 subjects were included. A meta-analysis was performed with STATA15.0 to estimate the combined standardized mean difference (SMD) with a 95% confidence interval (95% CI). Heterogeneity test of articles was examined by Q-test, and the results showed that *P* values of feed conversion ratio, average daily gain (ADG), and average daily intake were all less than 0.05, suggesting a strong heterogeneity among the selected literature. Therefore, the random effect model is selected to calculate the SMD of the three indexes. The combined SMDs (95% CI) of feed:gain, ADG, and average daily feed intake (ADFI) were −0.39 (−1.03, 0.25), 0.26 (−0.29, 0.81), and −1.45 (−3.09, 0.20), respectively, and the *P* values were all less than 0.05. This study shows that the absolute differences in the growth performance (feed:gain, ADG, and ADFI) of broilers fed with either organic or inorganic Se supplements at the same dose were quite small. The *P* values of Egger’s test were 0.770, 0.089, and 0.426, respectively, for the above indexes, showing no significant publication bias. Sensitivity analysis ensured the stability and reliability of the results. In summary, the effects of organic and inorganic Se in feed on the growth performance of broilers are statistically equal.

## 1 Introduction

Selenium (Se) is one of the substantial trace elements for animals, first discovered by Swedish chemist Berzelius in 1817. Se is an essential component of glutathione peroxidase, which has strong oxidation and immunity enhancing ability, and prevents some diseases in animals [1]. It also promotes growth and plays an important role in maintaining normal development and production of animals [2]. Se deficiency in poultry can lead to symptoms such as oozing diathesis, pancreatic fiber degeneration, and muscle nutritional atrophy, as well as phenomena such as reduced reproductive performance, thyroid dysfunction, decreased immune function, and reduced stress tolerance [3]. Se deficiency will lead to Keshan disease, Kashin–Beck disease, hypothyroidism, and a weakened immune system. Se has many such functions, which have become increasingly evident. Besides meeting basic nutritional requirements, it has potential health benefits [4].

Most areas of China are Se-deficient areas. Generally, Se in raw feed materials alone is not enough to meet the needs of animals for health and development, which should be supplemented by external sources [5]. Food is the main source of Se. The concentration of Se in foodstuffs is directly determined by the content of Se in the soil. An Se deficit in animals may be attributed to low Se levels in the soil. This mainly applies to cattle and sheep, with their direct link to soil via roughage. The risk of Se deficit in pigs and poultry is relatively lower because of reduced dependence on soil Se levels in local region and Se fortification in feed mixes [6]. Se deficiency is rare in most countries. However, it is quite common in China because of the soil type. Se can enter the food chain through plants, and through livestock and poultry products supplemented with Se additives. However, the variability in Se content of feedstuffs from different geographic regions makes Se supplementation of livestock and poultry diets an important safety factor in diet formulation [7]. Therefore, there are two benefits of Se supplementation for livestock and poultry: it can not only improve the health and performance of animals but also affect the quality of animal products (e.g., meat, milk, and eggs) and thereby improve human health [8]. Se is not only a nutritive functional additive but also a highly toxic mineral element. In China, the limit of Se in mixed feed for poultry is 0.5 mg/kg [9].

Se can be simply divided into organic and inorganic sources. Inorganic Se is passively absorbed in animal body in various ways, including simple diffusion absorption of selenite, coordinated transport of Se acid salt and sodium ions into the bloodstream in reponse to selenide deficiency, and transportation of cystine or sulfur compounds form loose structure to become a part of Se library [10]. Inorganic Se is mainly used in the form of sodium selenite, which is most widely used in livestock diets as a Se source. However, its strong toxicity, low bioavailability, and oxidation potential have adverse effects on animals and the environment [11]. Organic Se predominantly exists in the form of Se yeast or selenomethionine and has good absorption and utilization rates. It is absorbed by the body actively like an amino acid.

Selenomethionine is absorbed in small intestine by neutral amino acid transport system in animals. It shares amino acid transport mechanism of protein synthesis with methionine. It has been reported that the utilization rate of organic Se is higher than that of inorganic Se in laying hen diets because of its high bioavailability [12–13]. Organic Se generally exists in the form of Se yeast, which can be directly absorbed, converted, and used by animals [14–15]. It improves their antioxidant capacity, production performance, immunity, and anti-stress capacity and promotes body growth and development [16]. The absorption and metabolism of different forms of Se are quite different in the body [17]. For broilers, if supplemented with organic Se (Se yeast), the water loss of chicken can be greatly reduced, the quality of feathers can be significantly improved, and the production performance can also be improved by 2–4%. After being supplemented with Se yeast, the growth rate of broilers was also improved to a certain extent [18].

There is a lot of literature about the effects of organic and inorganic Se on the growth performance of broilers in China; however, the results differ, and there is a lack of randomized controlled trials (RCTs) with large sample sizes. To further elucidate the effects of different Se sources on the growth performance of broilers, this study identified and integrated previous studies on the effects of different Se sources on the growth performance of broilers for meta-analysis to provide a scientific basis for whether organic Se can replace inorganic Se as a nutritional additive.

## 2 Materials and methods

### 2.1 Materials

#### 2.1.1 Retrieval strategy

China National Knowledge Infrastructure (CNKI; https://www.cnki.net/), Wanfang (http://www.wanfangdata.com.cn/index.html), and Chinese Scientific Journal (VIP; http://www.cqvip.com/) databases were searched. Chinese search terms included “growth performance of broiler,” “production performance of broiler,” “growth of broiler,” “different selenium sources,” “organic selenium,” and so on. Literature that did not meet the inclusion criteria was manually excluded, with the reasons for exclusion indicated.

#### 2.1.2 Inclusion and exclusion criteria

Inclusion criteria were as follows: (1) the type of study was an RCT; (2) the research subject was broilers in China; (3) the intervention measures were adding organic Se to the diet of the experimental group and inorganic Se to the diet of the control group. The amount of Se additive was 0.3 mg/kg; and (4) the outcome index is the growth performance of broilers, including the feed conversion ratio, average daily gain (ADG), and average daily feed intake (ADFI) indicators. The mean value and standard deviation should be clearly stated in the study.

Exclusion criteria were as follows: (1) reviews, systematic evaluations, and repetitive literature; (2) studies inconsistent in their research content, intervention measures, and control measures; (3) non-RCT experiments and studies were not strictly prohibited on the basis of experimental design and method; (4) studies with inconsistent observation indexes, incomplete data, and inconsistent control; and (5) studies on Se combined with other mineral elements or probiotics.

### 2.2 Method

#### 2.2.1 Analysis method

In this study, meta-analysis was used to summarize and analyze multiple studies of different Se sources on the growth performance of broilers. At present, the meta-analysis of RCTs is common, and the relevant statistical analysis methods are relatively mature. Meta-analysis is suitable for large-scale and long-term studies when studies have shown different results, when decision-making is urgently required, and when there is a lack of time or inadequate conditions to carry out further experiments. By summarizing a large number of research results, meta-analysis can obtain results that are more persuasive than any single study and provide a good basis for drawing conclusions for urgent decision-making.

#### 2.2.2 Statistical analysis

Using Stata 15.0 software, the comprehensive effect estimate of each study was calculated. The comprehensive effect quantity is expressed by the standardized mean difference (SMD). The SMD can eliminate the influence of different research measurement units. The Q-test was used to test whether there was heterogeneity among the studies, and Egger’s regression test was used to test whether there was bias in literature publication.

## 3 Results

### 3.1 Literature search results

**Figure 1:**
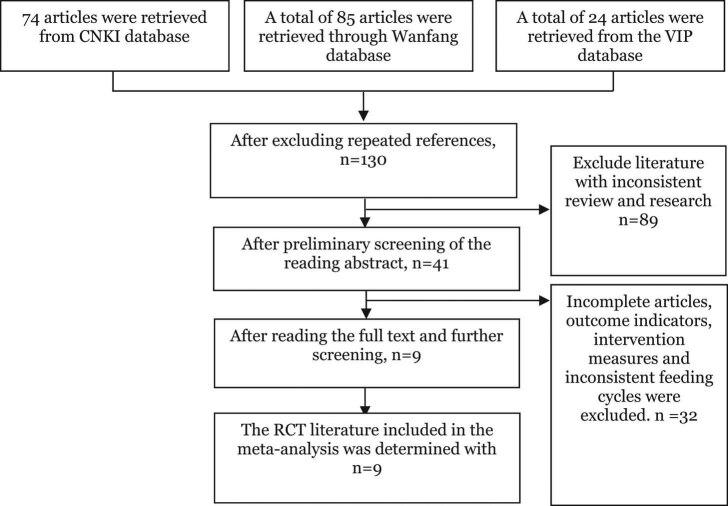
Literature retrieval process.

In this study, 183 RCTs were retrieved from the databases (74 from China CNKI, 85 from Wanfang, and 24 from VIP). Of them, 130 were left after duplicate exclusion. Another 89 RCTs were excluded by the preliminary screening of titles and abstracts, and 32 after reading full articles. Ultimately, nine studies were included in this meta-analysis in line with the aforementioned inclusion and exclusion criteria. The included literature was published between 2001 and 2016, among which the number of studies corresponding to the topics of feed conversion ratio, ADG, and average daily intake was nine, nine, and six [19,20,21,22,23,24,25,26,27], respectively. The retrieval flow chart is shown in Figure 1.

### 3.2 Document content extraction

The content of the literature that met the inclusion criteria was extracted, which included the following: (1) the first author and published years; (2) the sample size of the control group and the experimental group, the intervention measures of the experimental group, and the experimental period; (3) the source of Se; and (4) the outcome indicators (feed conversion ratio, ADG, mean, and standard deviation of ADFI) (Table 1).

**Table 1:** Basic characteristics of the included studies

First author	Publication year	Number of samples	Test cycle time/day	Types and sources of organic Se	Index value (A: inorganic Se, B: organic Se)		
					Feed:gain	ADG/g	ADFI/g
Xiaojing Yang	2001	30	12	Se yeast (American Otchi Company)	A = 2.20 ± 0.08	A = 57.70 ± 1.68	
					B = 2.26 ± 0.04	B = 59.56 ± 1.41	
Feng Guo	2004	45	49	Se yeast	A = 2.48 ± 0.11	A = 53.47 ± 2.04	A = 132.38 ± 0.86
					B = 2.22 ± 0.12	B = 55.43 ± 2.01	B = 123.05 ± 1.83
Feili Xu	2007	24	28	Selenomethionine (Sichuan Chengdu Xu Health Products Technology Co. Ltd)	A = 1.73 ± 0.02	A = 18.16 ± 0.35	A = 31.42 ± 8.92
					B = 1.70 ± 0.01	B = 19.83 ± 2.07	B = 33.71 ± 3.54
Qinghua Song	2009	68	53	Methionine Se	A = 2.48 ± 0.08	A = 26.73 ± 2.25	
					B = 2.62 ± 0.14	B = 26.63 ± 0.63	
Chun Fan	2009	24	27	Se yeast	A = 2.04 ± 0.11	A = 56.31 ± 3.15	A = 116.06 ± 3.70
					B = 2.03 ± 0.02	B = 57.14 ± 0.69	B = 116.84 ± 1.93
Sifeng Yi	2010	30	35	Se yeast (Angel Yeast Co. Ltd)	A = 1.94 ± 0.05	A = 57.82 ± 1.21	A = 121.74 ± 1.43
					B = 1.93 ± 0.06	B = 58.00 ± 1.14	B = 121.77 ± 1.41
Jinke Tian	2012	120	42	Se yeast (Wuhan Xinhua Yang Biological Co. Ltd)	A = 1.76 ± 0.03	A = 53.79 ± 0.98	A = 94.27 ± 0.70
					B = 1.73 ± 0.03	B = 52.75 ± 0.43	B = 91.01 ± 1.22
Junrui Guo	2014	108	42	Se yeast	A = 1.81 ± 0.05	A = 52.80 ± 2.20	A = 95.50 ± 5.70
					B = 1.81 ± 0.06	B = 53.60 ± 3.00	B = 96.70 ± 3.50
Xue Xiao	2016	90	21	Se yeast	A = 1.58 ± 0.08	A = 22.19 ± 0.79	
					B = 1.56 ± 0.05	B = 22.11 ± 0.02	

### 3.3 Meta-analysis of the effect of different Se sources on growth performance of broilers

#### 3.3.1 Heterogeneity test

**Figure 2:**
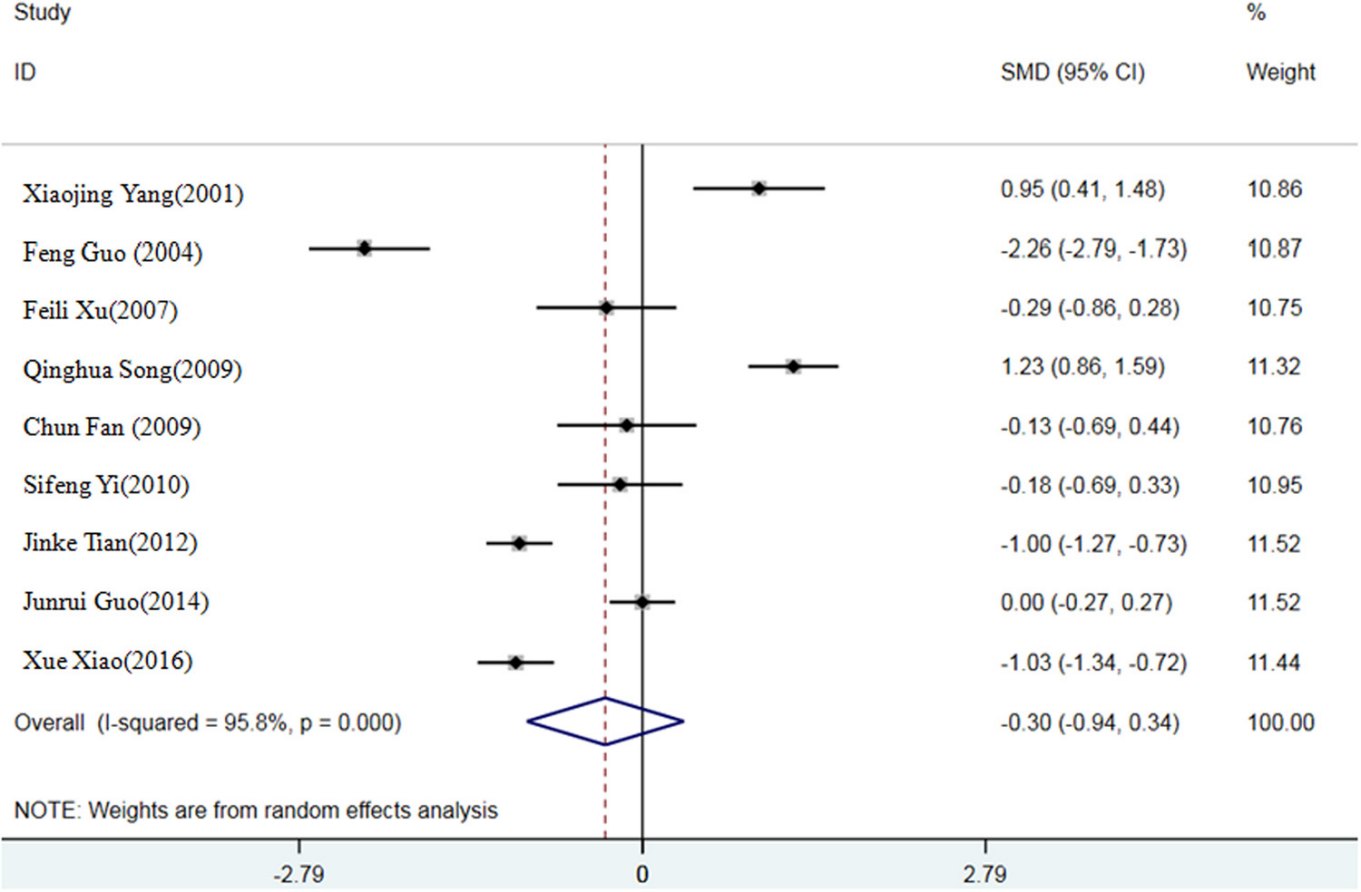
A meta-analysis forest map of effects of different Se sources on feed conversion ratio of broilers.

**Figure 3:**
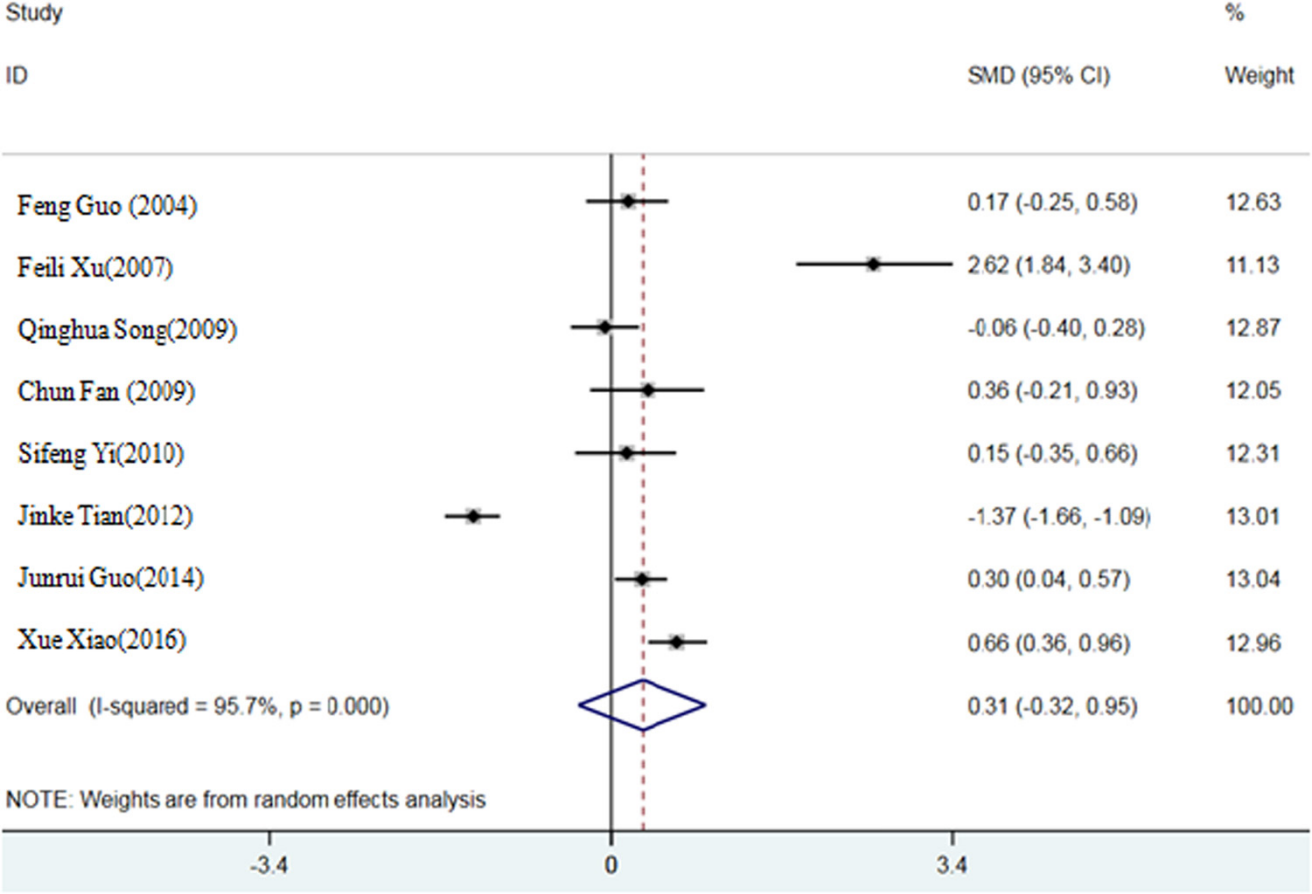
A meta-analysis forest map of effects of different Se sources on average daily weight gain of broilers.

**Figure 4:**
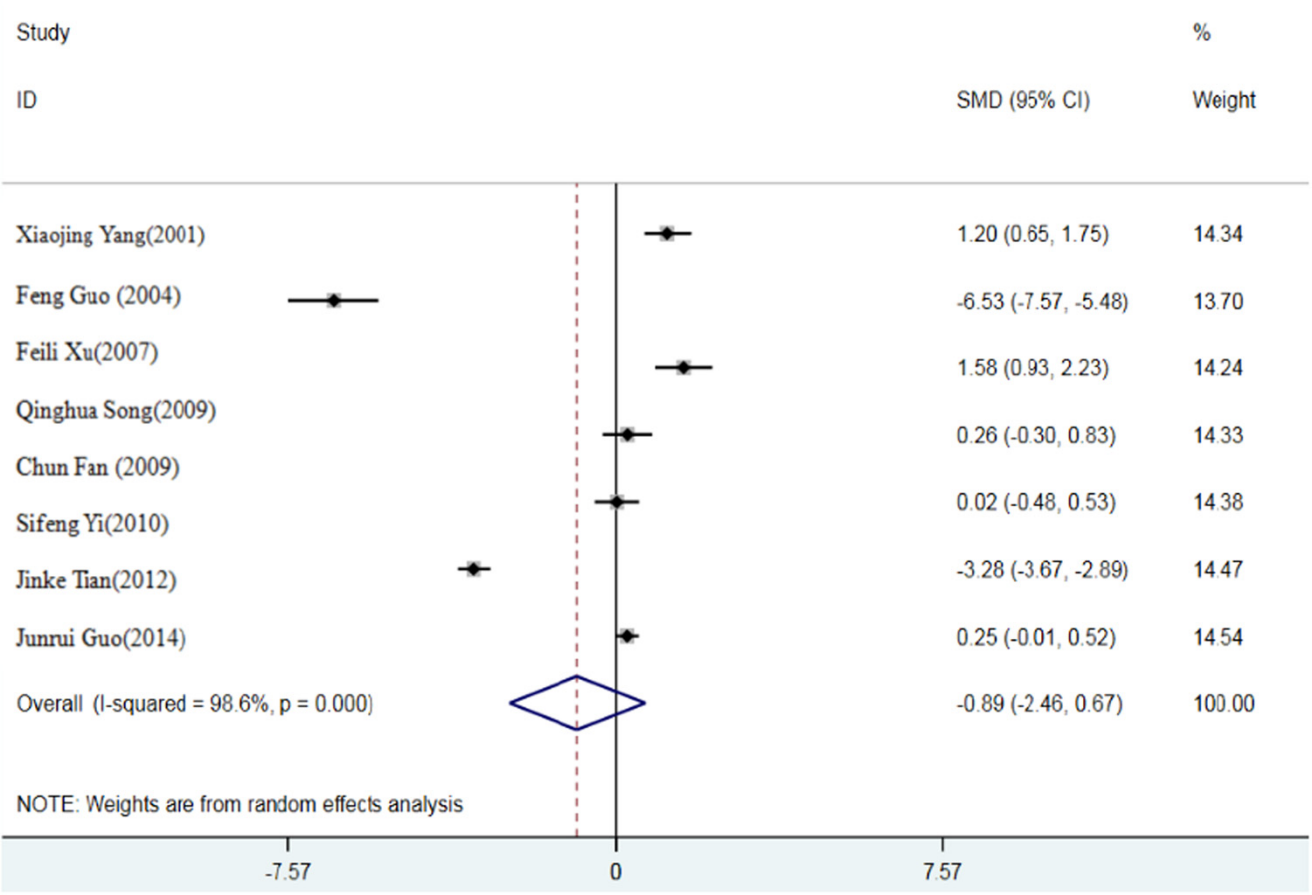
A meta-analysis of forest map of effects of different Se sources on daily feed intake of broiler.

The results of Q-test showed that the *p* values of feed conversion ratio, ADG, and ADFI were all less than 0.05, suggesting a strong heterogeneity among the selected literature in this study. Therefore, the random effect model is selected to calculate the SMD of the three indexes. Compared with the inorganic Se control group, the SMD of the organic Se experimental group was −0.39, 95% confidence interval (95% CI: −1.03, 0.25) (*P* < 0.05) (Figure 2); SMD of ADG was 0.26, 95% CI (−0.29, 0.81) (*P* < 0.05) (Figure 3); and SMD of ADFI was −1.45, 95% CI (−3.09, 0.20) (*P* < 0.05) (Figure 4).

#### 3.3.2 Publication bias test

The bias test was carried out on the literature included in the study. Because less than ten studies were included in the study, Egger’s regression test was used for analysis. The *p* values of Egger’s test were 0.770, 0.089, and 0.426 for feed conversion ratio, ADG, and ADFI, respectively. All the *p* values were greater than 0.05, showing no significant publication bias within the three study topics.

#### 3.3.3 Sensitivity analysis

To test the sensitivity of the research results, the method of maximum weight measurement was applied in the study of feed conversion ratio, ADG, and ADFI. It is visible that the two maximum weighted measurements for the feed conversion ratio were −0.31 (−1.01, 0.40) and −0.44 (−1.21, 0.33), respectively, after removing the maximum weighted measurement; the combined SMD for ADG was 0.26 (−0.39, 0.91) after the removal of the maximum weight; and the combined SMD for average daily intake was −1.80 (−3.88, 0.27) after the removal of the most important weight. It can be seen that there is no significant change in the combined SMD of the three indexes after the removal of the largest weighted measurement, which indicates that the sensitivity of the included literature is relatively small and the research results were stable and reliable.

## 4 Discussion

So far there were plenty of studies on the effects of organic and inorganic Se on the growth performance of chickens, but the results were inconsistent because of different research periods, sample sizes, growth stages, growth environments, or feed conditions of the research subjects. Current research conducted a meta-analysis of previous studies and found that the effects of organic and inorganic Se in feed are statistically the same on the growth performance of broilers. Egger’s test did not point out any significant bias in the publication of literature. In the sensitivity analysis, there was no significant change in the combined SMD when the largest weight measurement of the three indexes was removed, indicating that the sensitivity was low, and the research results were reliable.

At present, there are great differences in the published research results of the effects of organic and inorganic Se on the growth performance of broilers. Xu [23] found that the growth performance of 49-day yellow-feathered broiler with 0.60 mg/kg organic Se has been superior. Bakhshalinejad et al. [28] found that feeding a diet containing 0.4 mg/kg of Se regardless of its source results in greatest ADG [23]. Zou et al. and Li et al. found that the effect of 0.15 mg/kg organic Se on growth performance of broilers was significantly improved than inorganic Se, and the growth promoting effect of sodium selenite is not obvious, which may be related to the low level of sodium selenite [29,30]. On the contrary, the studies of Tian et al. [20] and Song and Tian [21] showed that the ADG and feed conversion ratio of broilers supplemented with different Se sources had no significant differences. The different results in different articles may be related to broiler breeds (Tian et al. [20] used AA broilers and Song and Tian [21] used Ma Yu broiler, a local broiler breed in Guangxi province, China), test period, test environment, feeding conditions, and other factors, which need to be further studied.

The price of inorganic Se is low, which is an economic advantage for farmers. However, the toxicity of inorganic Se is high, which may damage the livers of broilers and reduce the quality of meat. The utilization rate is low and the unused inorganic Se may pollute the environment. The price of organic Se is high, but it can be directly absorbed and used by the small intestine. It has a relatively good utilization rate and therefore causes less environmental pollution [19]. In addition, the retention time of organic Se in chickens is longer than that of inorganic Se. In good nutritional condition, organic Se can be stored. In case of insufficient nutritional intake of Se in the organism, the stored organic Se can be replenished into physiological metabolism, so as to meet the body’s demand for Se. Organic Se can improve the body’s immunity and affect the growth performance of the organism by affecting its ingestion and absorption [1].

## 5 Conclusion

This meta-analysis summarized previous studies on the effects of different Se sources on the growth performance of broilers, and the results showed that there was no significant difference between the effects of organic and inorganic Se supplementation in broilers of China, which ended some controversies in this research field to a certain extent. Hence, farms can choose either organic or inorganic Se feed additives according to their own needs and conditions. Considering the growth performance of livestock only, there is no significant difference between these two sources of Se. However, considering the high utilization rate and environment-friendly characteristics of organic Se, we recommend using organic Se as an additive in broiler’s feed.
